# Supplementary figures and images for: Multi-omics and network pharmacology identify IGFBP1 as an m6A-Epigenetic target of pueraria in NSCLC therapy
Source: PLoS Comput Biol. 2026 Mar 12;22(3):e1014050. doi: 10.1371/journal.pcbi.1014050 (PMC12981440; doi:10.1371/journal.pcbi.1014050)

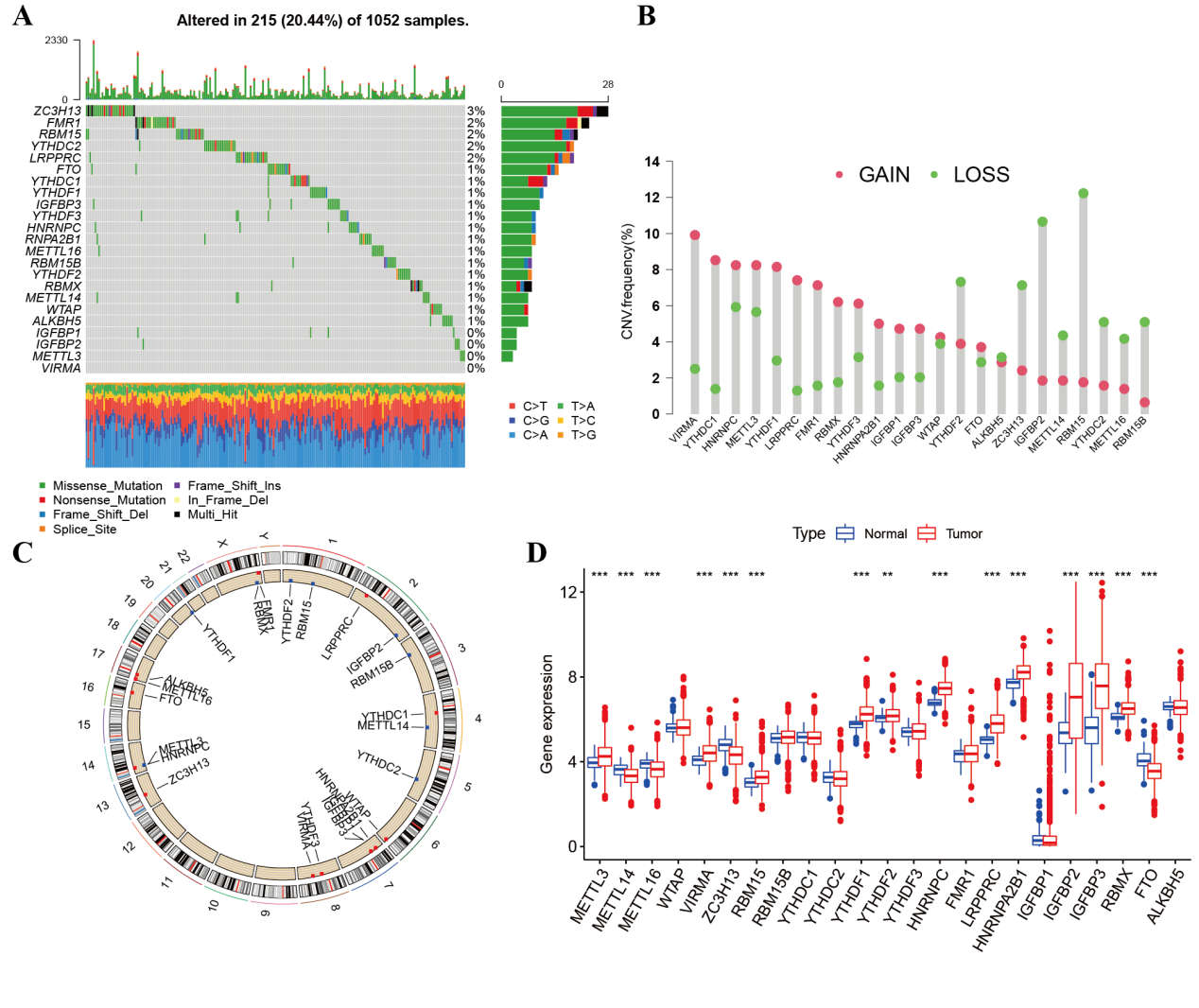

Supplement: S1 Fig — A The maftools of somatic mutation burden frequency of 23 m6A-associated modulators in NSCLC. B The copy number alteration frequency of 23 m6A-associated modulators in NSCLC. C The copy number circle graph of 23 m6A-associated modulators in 23 human chromosomes. D The differential expression level of 23 m6A-associated modulators in NSCLC and normal tissues. (TIF) [file pcbi.1014050.s001.tif]

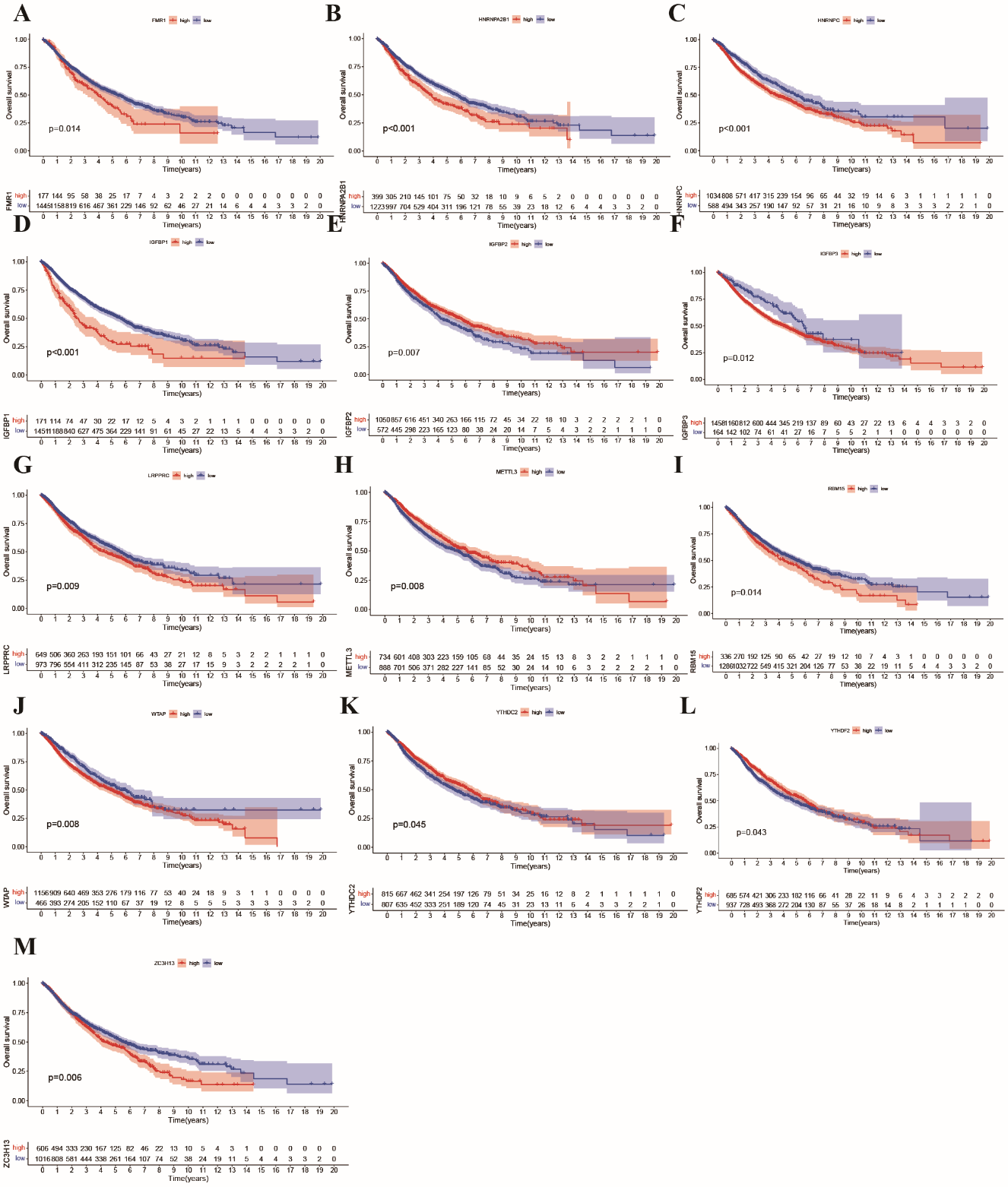

Supplement: S2 Fig — The red and blue lines reveal m6A regulators with high and low expression, respectively. A, B, C, D, F, G, I, J, M Survival analysis showed that high expression of FMR1, HNRNPA2B1, HNRNPC, IGFBP1, IGFBP3, LRPPRC, RBM15, WTAP, ZC3H13 have a poor prognosis compared with low expression of them. E, H, K, L Survival analysis showed that low expression of IGFBP2, METTL3, YTHDC2, YTHDF2 have a poor prognosis compared with high expression of them. (TIF) [file pcbi.1014050.s002.tif]

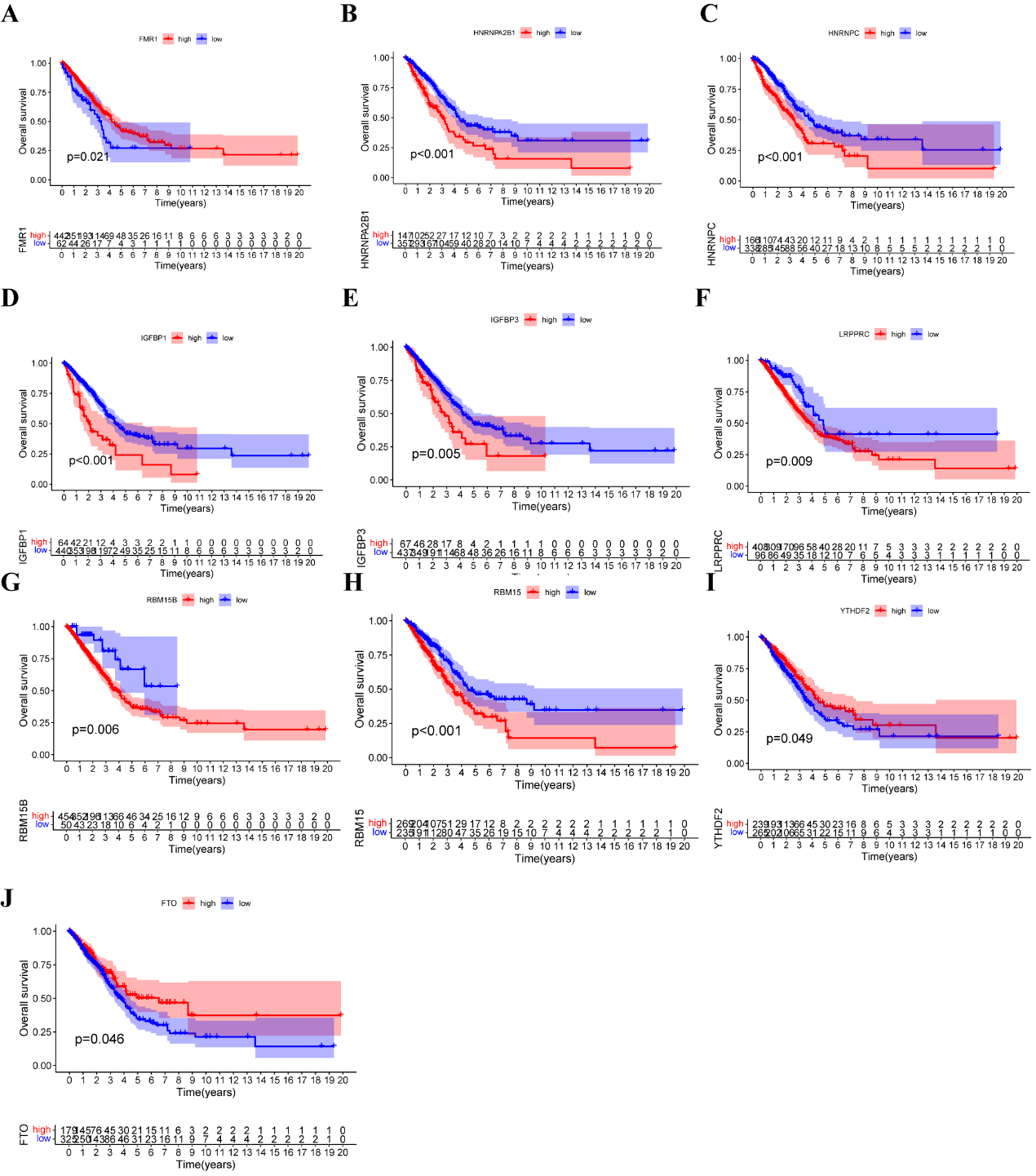

Supplement: S3 Fig — A, I, J Survival analysis showed that low expression of FMR1, YTHDF2, FTO have a poor prognosis compared with the high expression of them. B-H Survival analysis showed that high expression of HNRNPA2B1, HNRNPC, IGFBP1, IGFBP3, LRPPRC, RBM15B, RBM15 have a poor prognosis compared with the low expression of them. (TIF) [file pcbi.1014050.s003.tif]

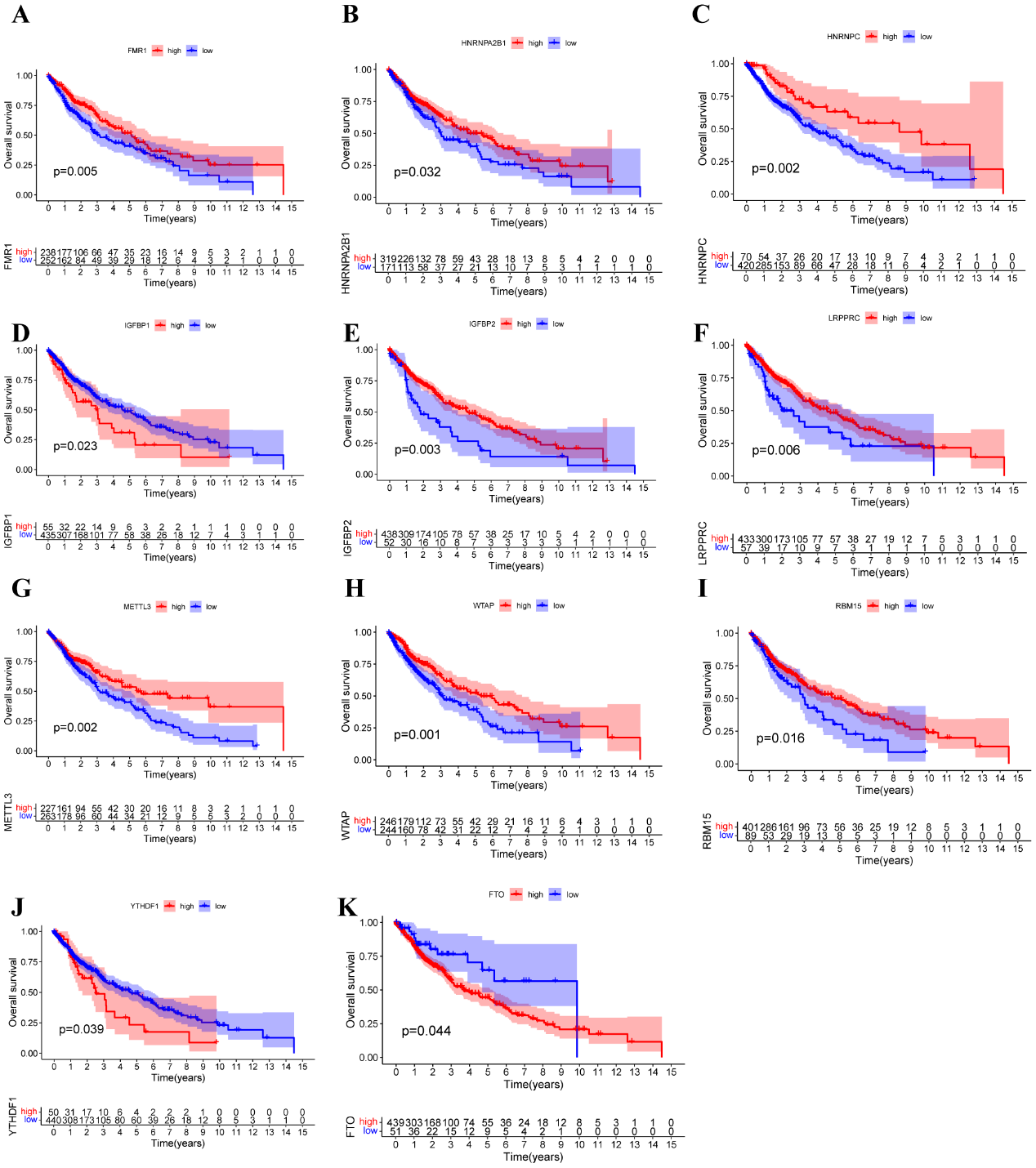

Supplement: S4 Fig — A, B, C, E, F, G, H, I Survival analysis showed that low expression of FMR1, HNRNPA2B1, HNRNPC, IGFBP2, LRPPRC, METTL3, WTAP, RBM15 have a poor prognosis compared with high expression of them. D, J, K Survival analysis showed that high expression of IGFBP1, YTHDF1, FTO have a poor prognosis compared with low expression of them. (TIF) [file pcbi.1014050.s004.tif]

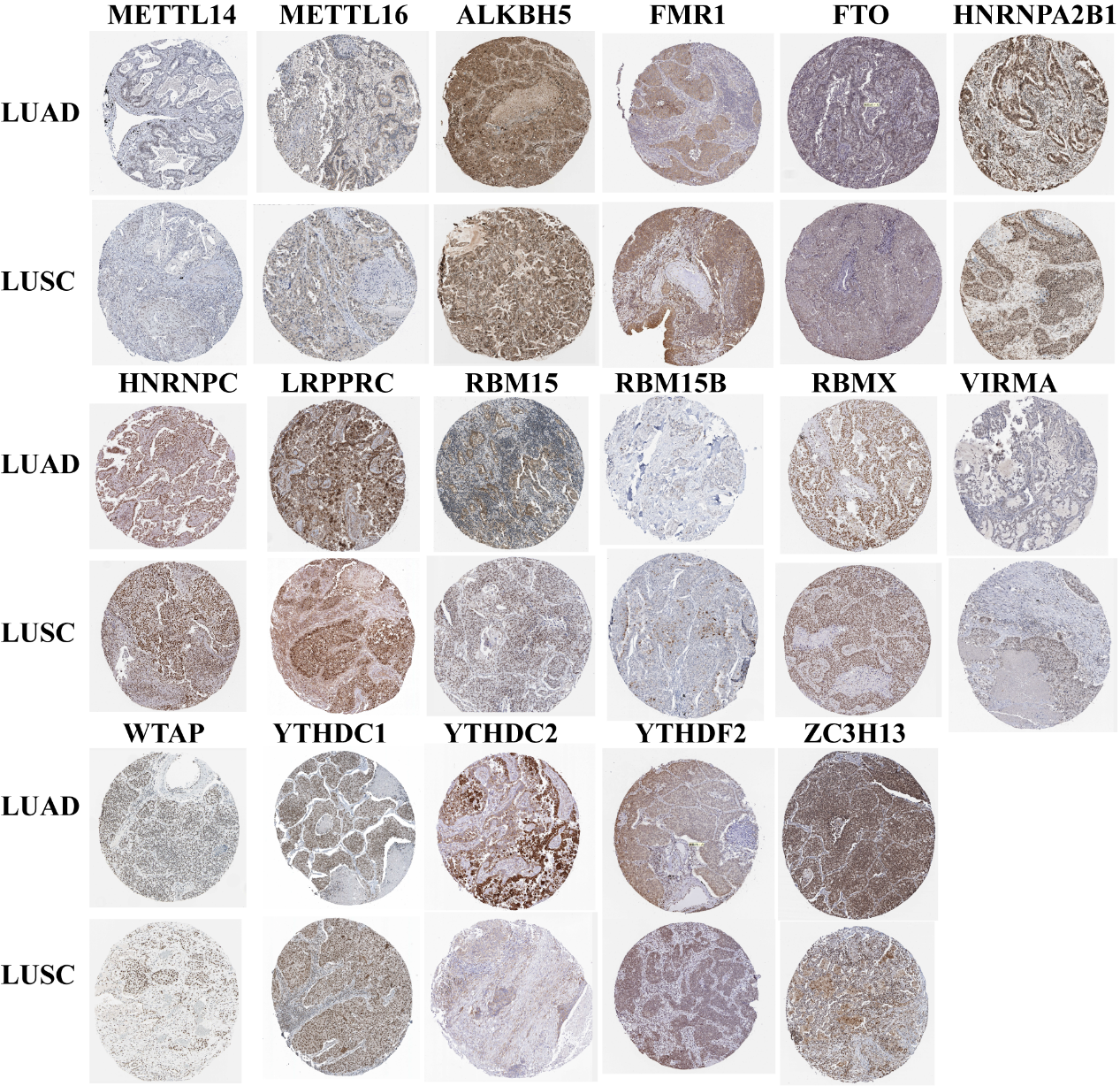

Supplement: S5 Fig — A-H Survival analysis focusing on clinical attributes among low-risk and high-risk groups of patients diagnosed with NSCLC. Patients with NSCLC who possess a high-risk score, particularly those aged over 65 or under 65, regardless of gender, as well as those classified with nodal involvement (N0 and N1-3) and tumor stages (T1-2 and T3-4), exhibit a poorer prognosis compared to their low-risk score counterparts. The red and blue lines represent cohorts categorized as high risk and low risk, respectively. (TIF) [file pcbi.1014050.s005.tif]

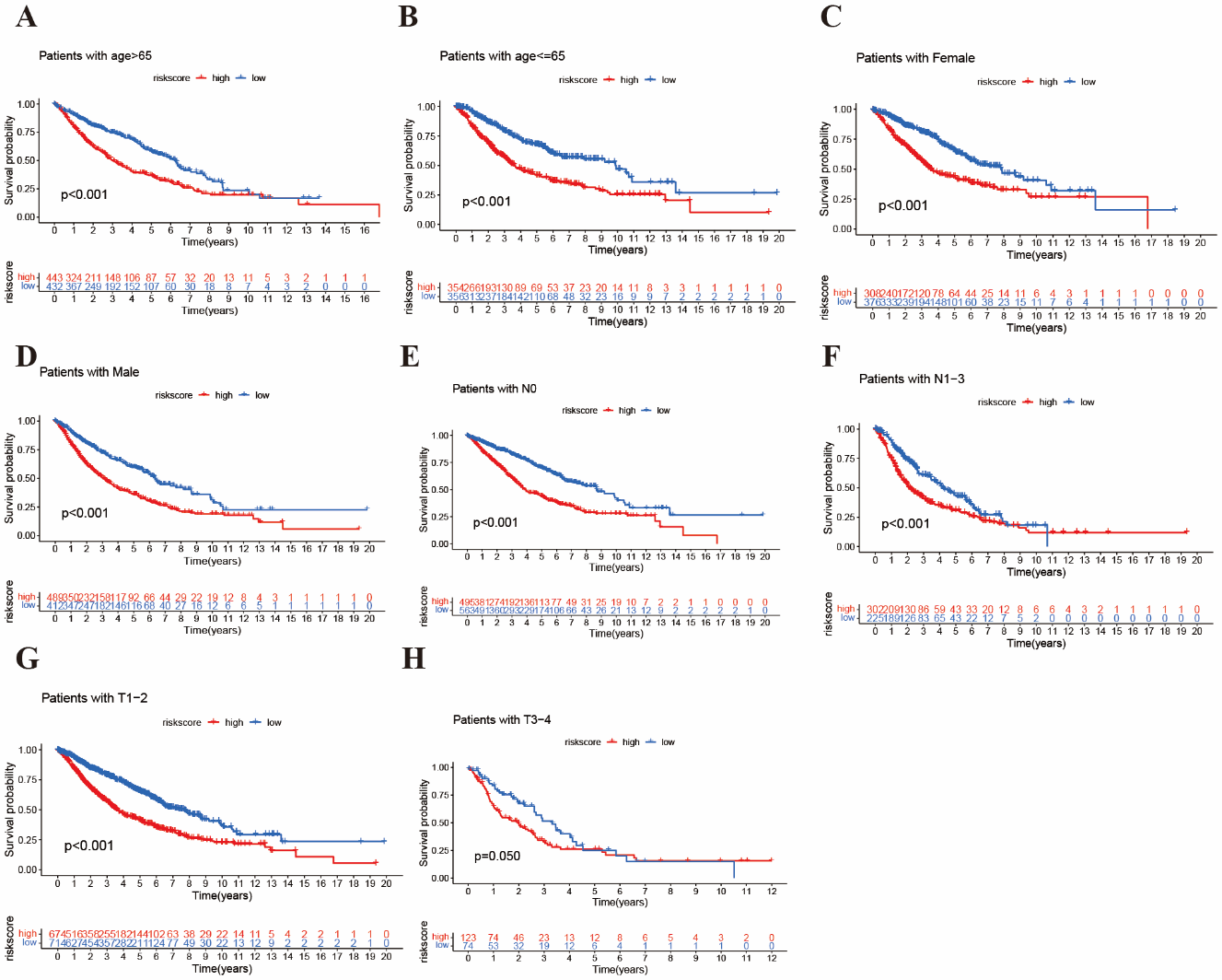

Supplement: S6 Fig — The protein expressions of ALKBH5, FMR1, FTO, HNRNPA2B1, HNRNPC, LRPPRC, RBM15, RBMX, YTHDC1, YTHDC2, YTHDF2, and ZC3H13 were strongly positive in LUAD and LUSC tissues, while the protein expressions of METTL14, METTL16, RBM15B, VIRMA, and WTAP were weakly positive in LUAD and LUSC tissues. (TIF) [file pcbi.1014050.s006.tif]

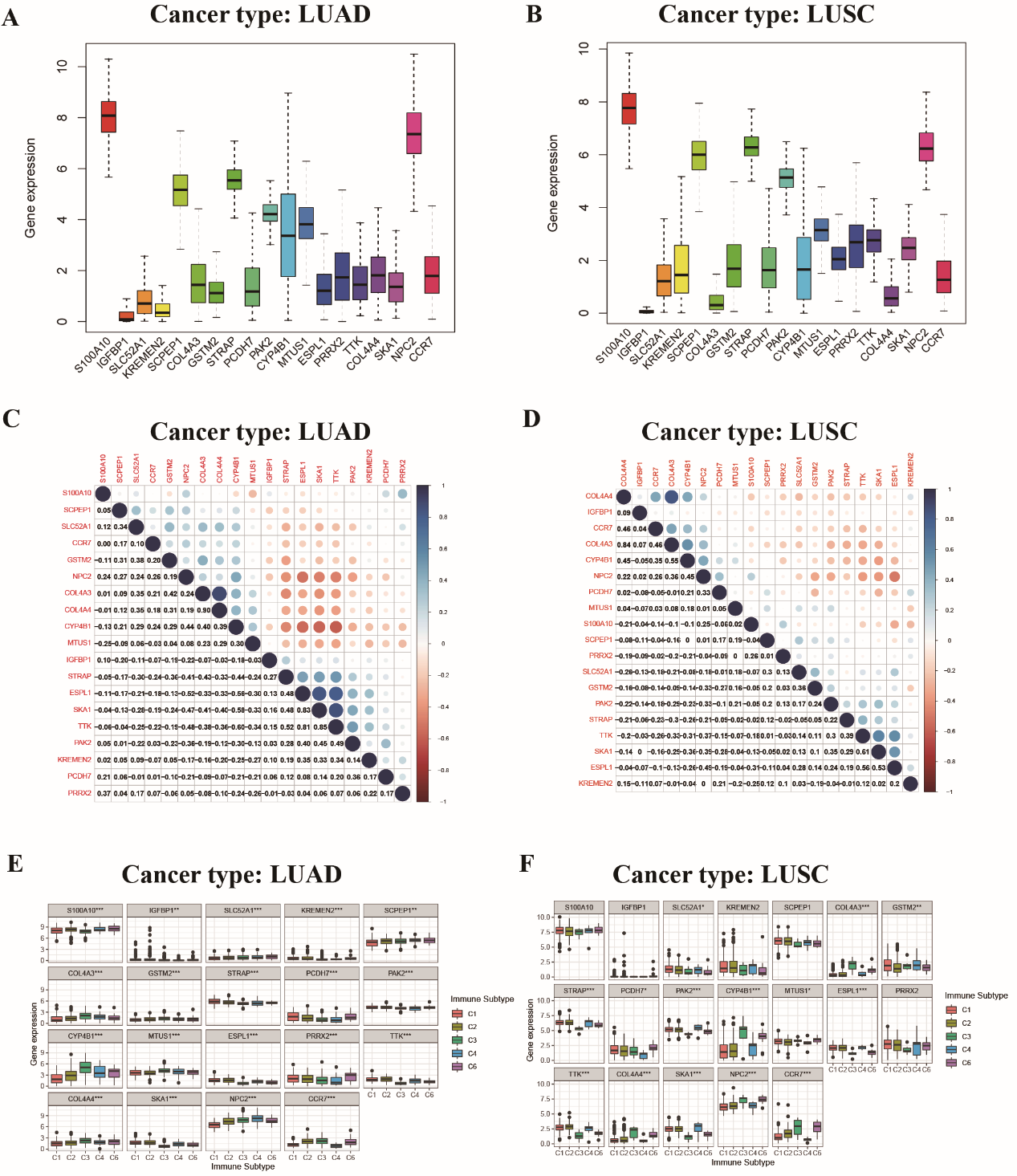

Supplement: S7 Fig — A-B Boxplot of the 19 prognostic m6A cluster differential genes in LUAD and LUSC tissues from UCSC-Xena. C-D Correlation analysis of the expression of the 19 prognostic m6A cluster differential genes in LUAD and LUSC. E-F The relationship between the expression of 19 m6A cluster differential genes and different immune subtypes. (TIF) [file pcbi.1014050.s007.tif]

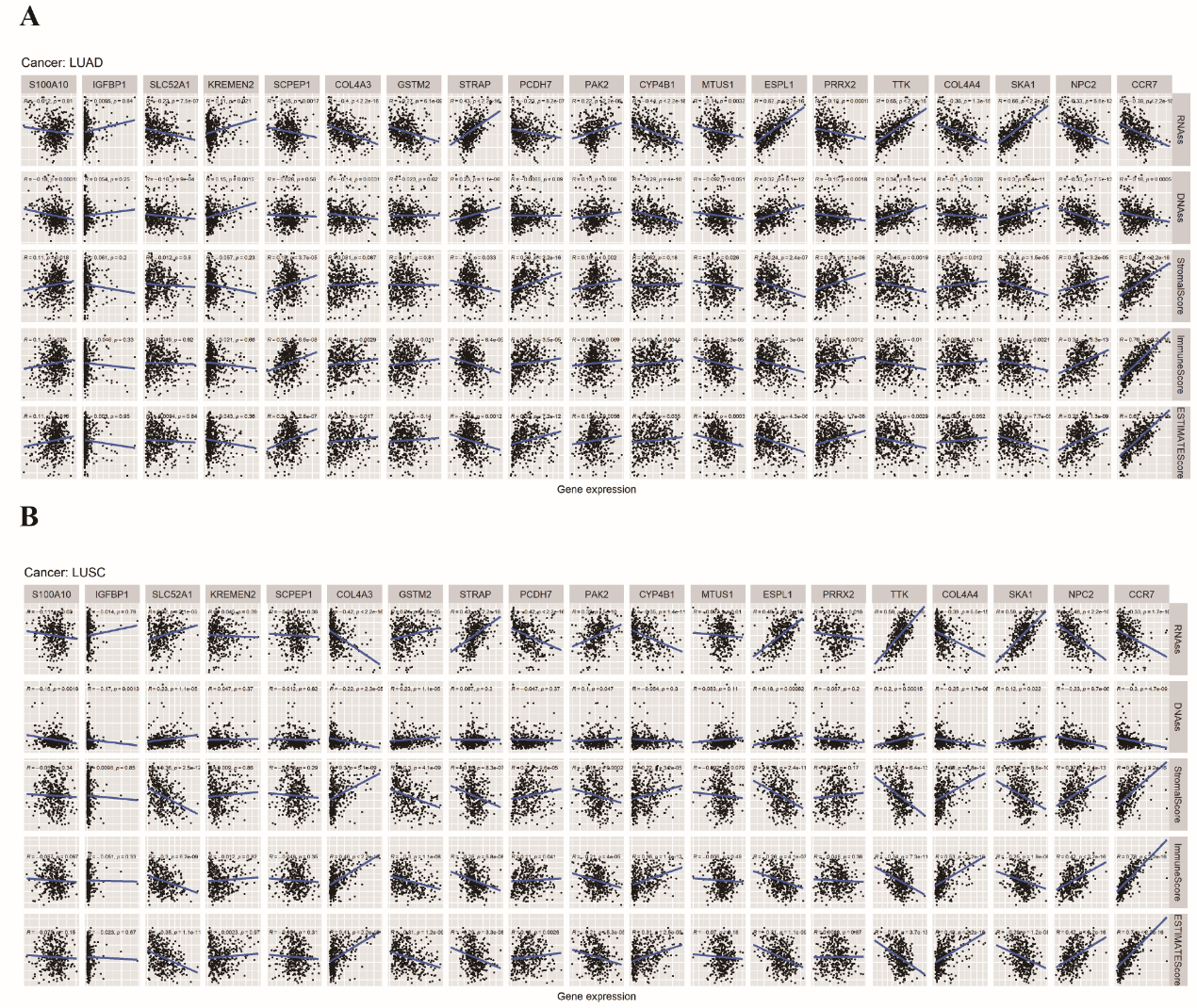

Supplement: S8 Fig — A The relationship between m6A cluster differential gene expression and stemness score, immune microenvironment in LUAD. B Association analysis of m6A cluster differential gene expression and stemness score, immune microenvironment in LUSC. (TIF) [file pcbi.1014050.s008.tif]

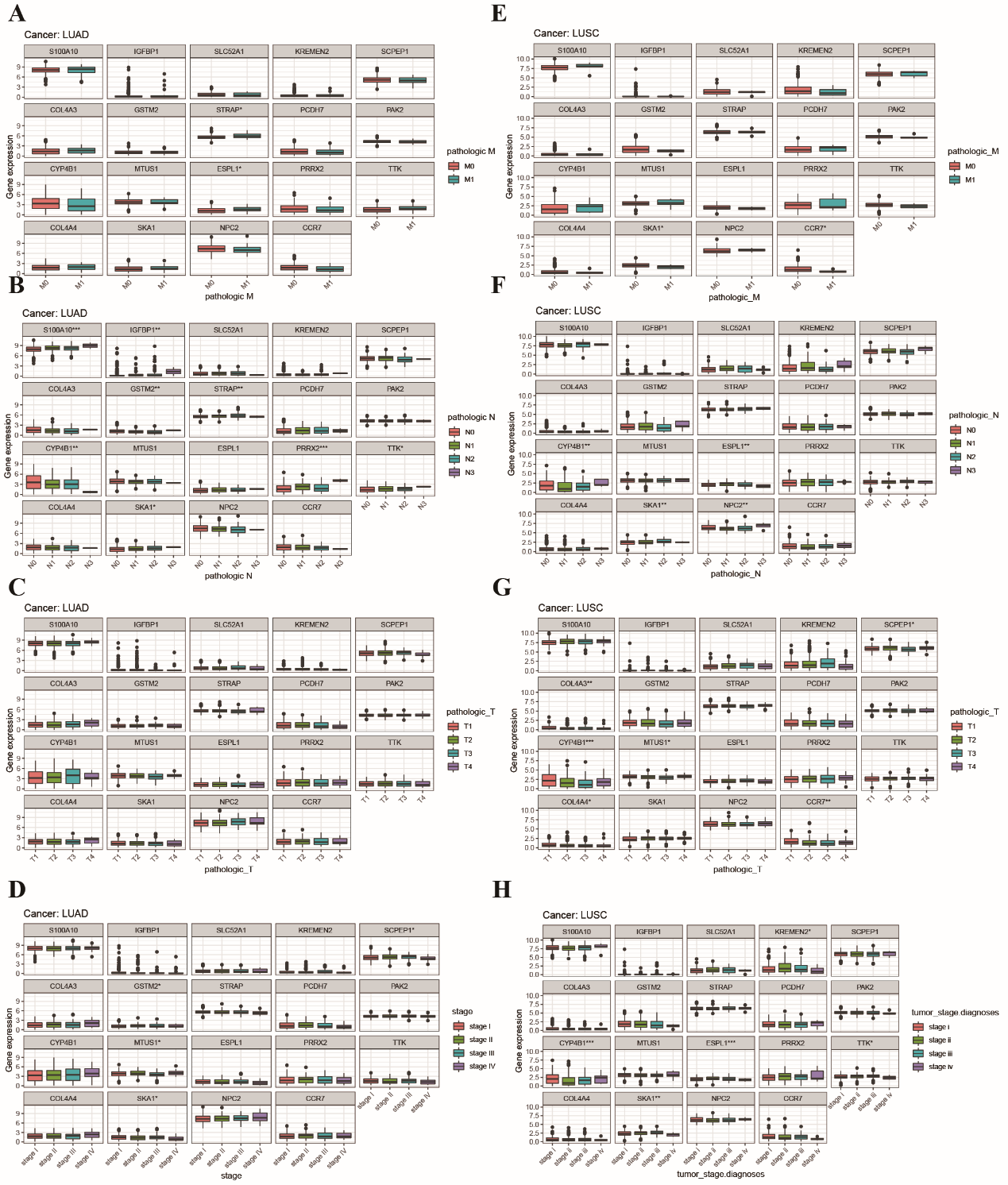

Supplement: S9 Fig — A-D The relationship between m6A cluster differential gene expression and clinical pathological features of LUAD patients. E-H Association analysis of m6A cluster differential genes expression and clinical pathological features of LUSC patients. (TIF) [file pcbi.1014050.s009.tif]

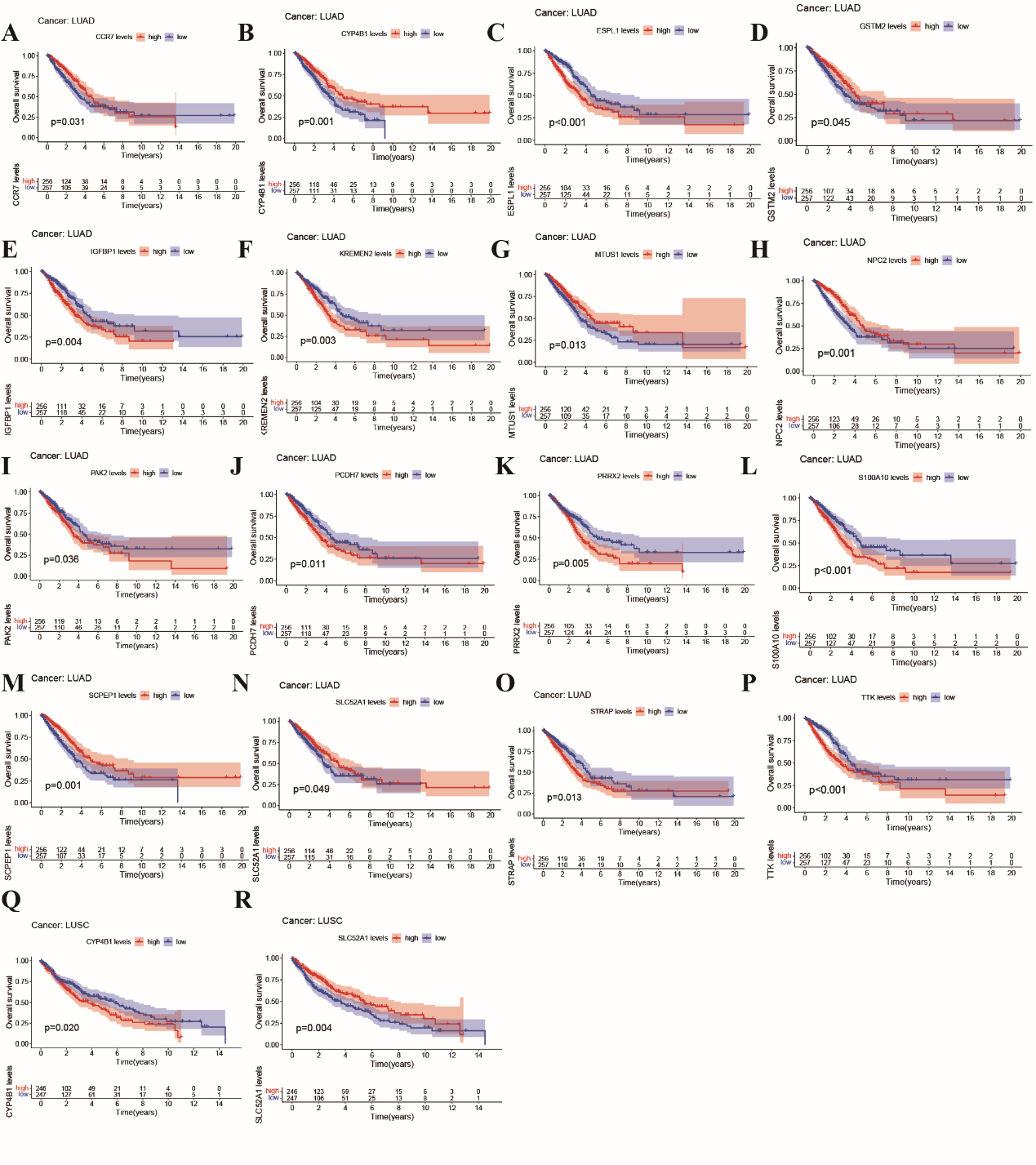

Supplement: S10 Fig — A-P Survival analysis of m6A cluster differential genes in LUAD. High expression of CCR7, CYP4B1, GSTM2, MTUS1, NPC2, SCPEP1, SLC52A1 had a better clinical outcome than low expression of them in LUAD patients. Low expression of ESPL1, IGFBP1, KREMEN2, PAK2, PCDH7, PRRX2, S100A10, STRAP, TTK had a better clinical outcome than high expression of them in LUAD patients. Q-R Survival analysis of m6A cluster differential genes in LUSC. High expression of CYP4B1 had a poorer prognosis than low expression of CYP4B1 in LUSC patients. High expression of SLC52A1 had a better clinical outcome than low expression of SLC52A1 in LUSC patients. (TIF) [file pcbi.1014050.s010.tif]

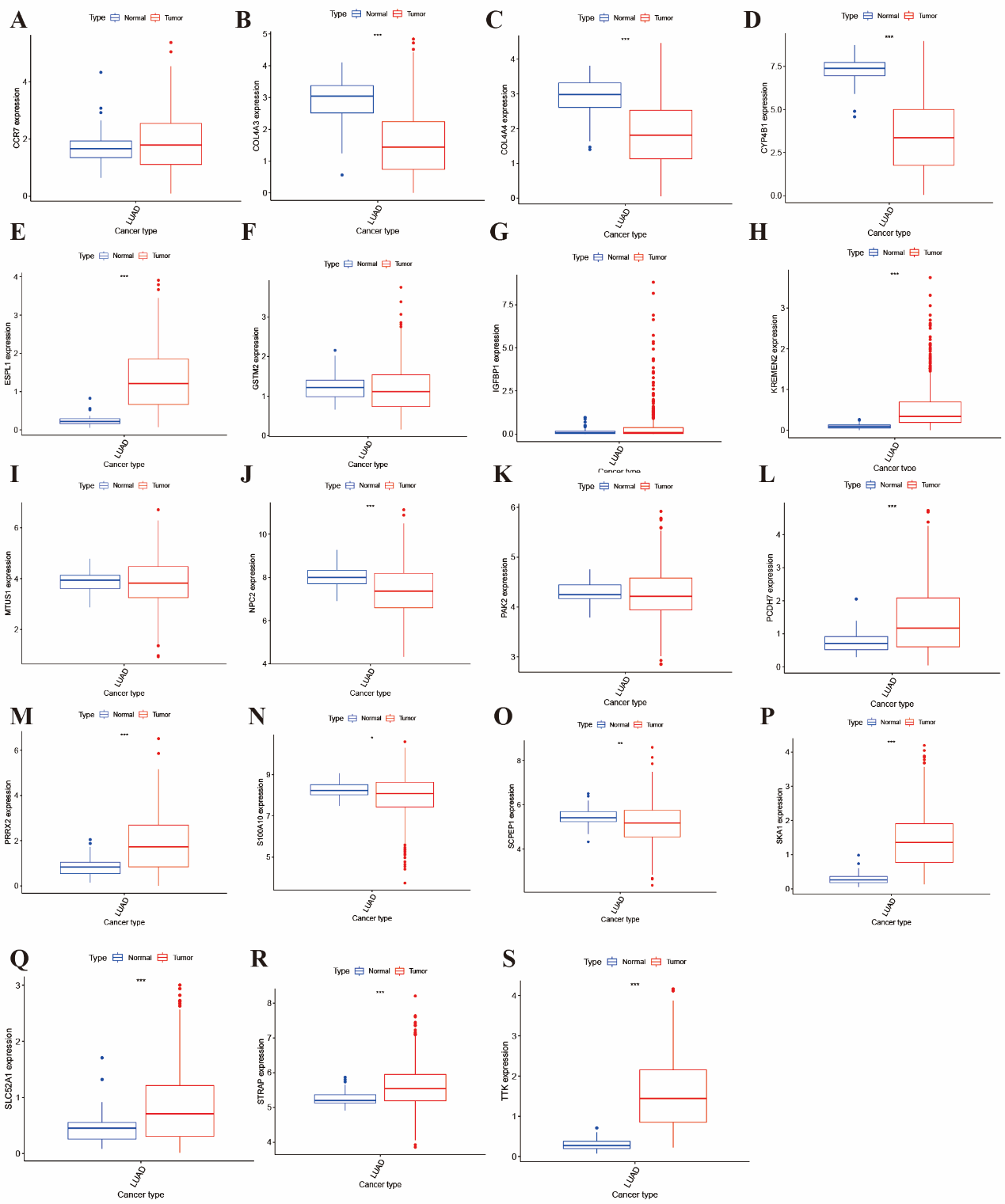

Supplement: S11 Fig — A-S Red and blue boxplots indicate LUAD and normal tissues in the TCGA database. CCR7, ESPL1, KREMEN2, NPC2, PAK2, PCDH7, PRRX2, S100A10, SCPEP1, SKA1, SLC52A1, STRAP, TTK were highly expressed in LUAD tissues. COL4A3, COL4A4, CYP4B1 were down-expressed in LUAD tissues. * Represents P < 0.05, ** represents P < 0.01, *** represents P < 0.001, **** represents P < 0.0001. (TIF) [file pcbi.1014050.s011.tif]

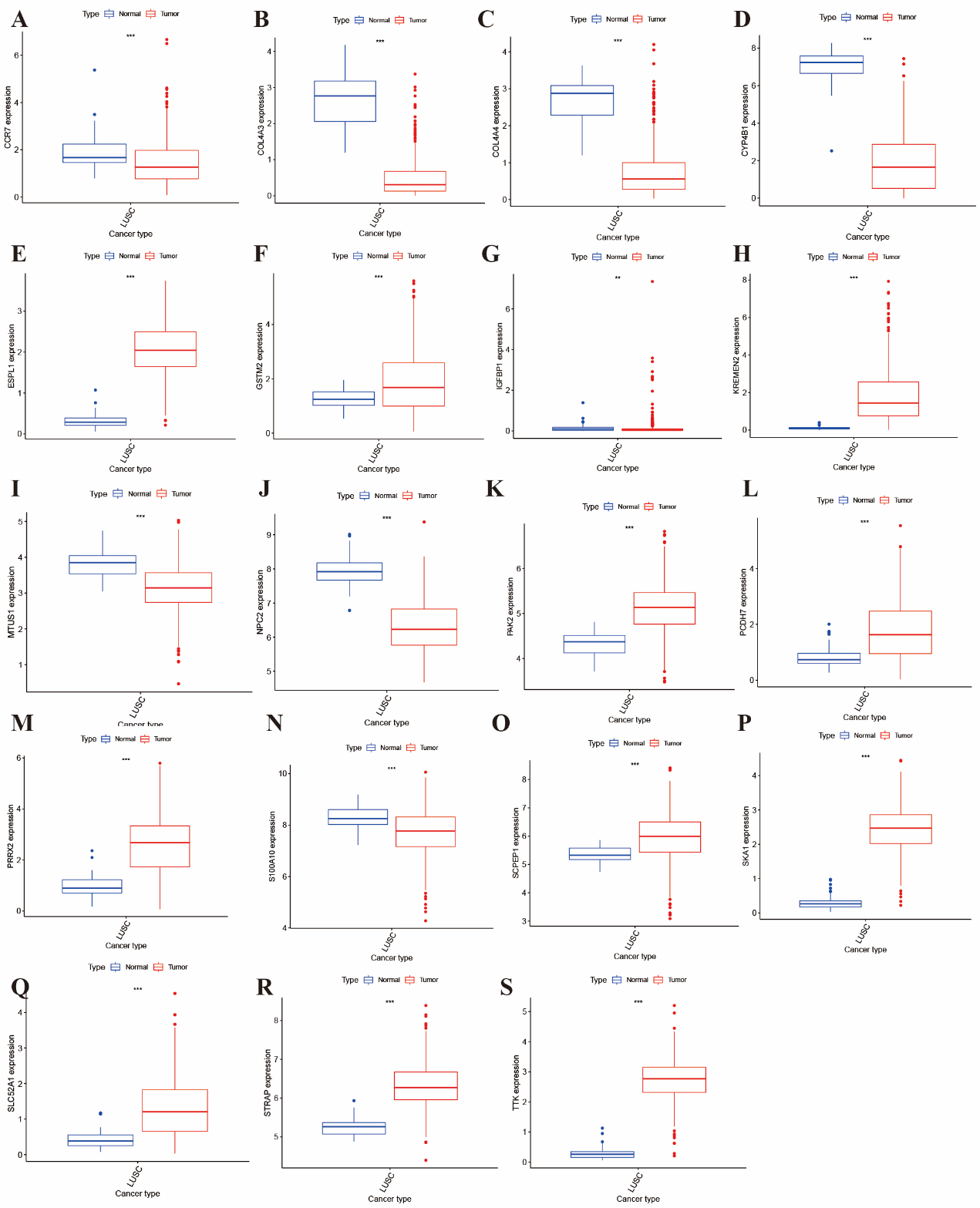

Supplement: S12 Fig — A-S Red and blue boxplots indicate LUSC and non-LUSC tissues from the TCGA database. CCR7, COL4A3, COL4A4, CYP4B1, MTUS1, NPC2, S100A10 were highly expressed in non-LUSC tissues. ESPL1, GSTM2, IGFBP1, KREMEN2, PAK2, PCDH7, PRRX2, SCPEP1, SKA1, SLC52A1, STRAP, TTK were highly expressed in LUSC tissues. * Represents P < 0.05, ** represents P < 0.01, *** represents P < 0.001, **** represents P < 0.0001. (TIF) [file pcbi.1014050.s012.tif]

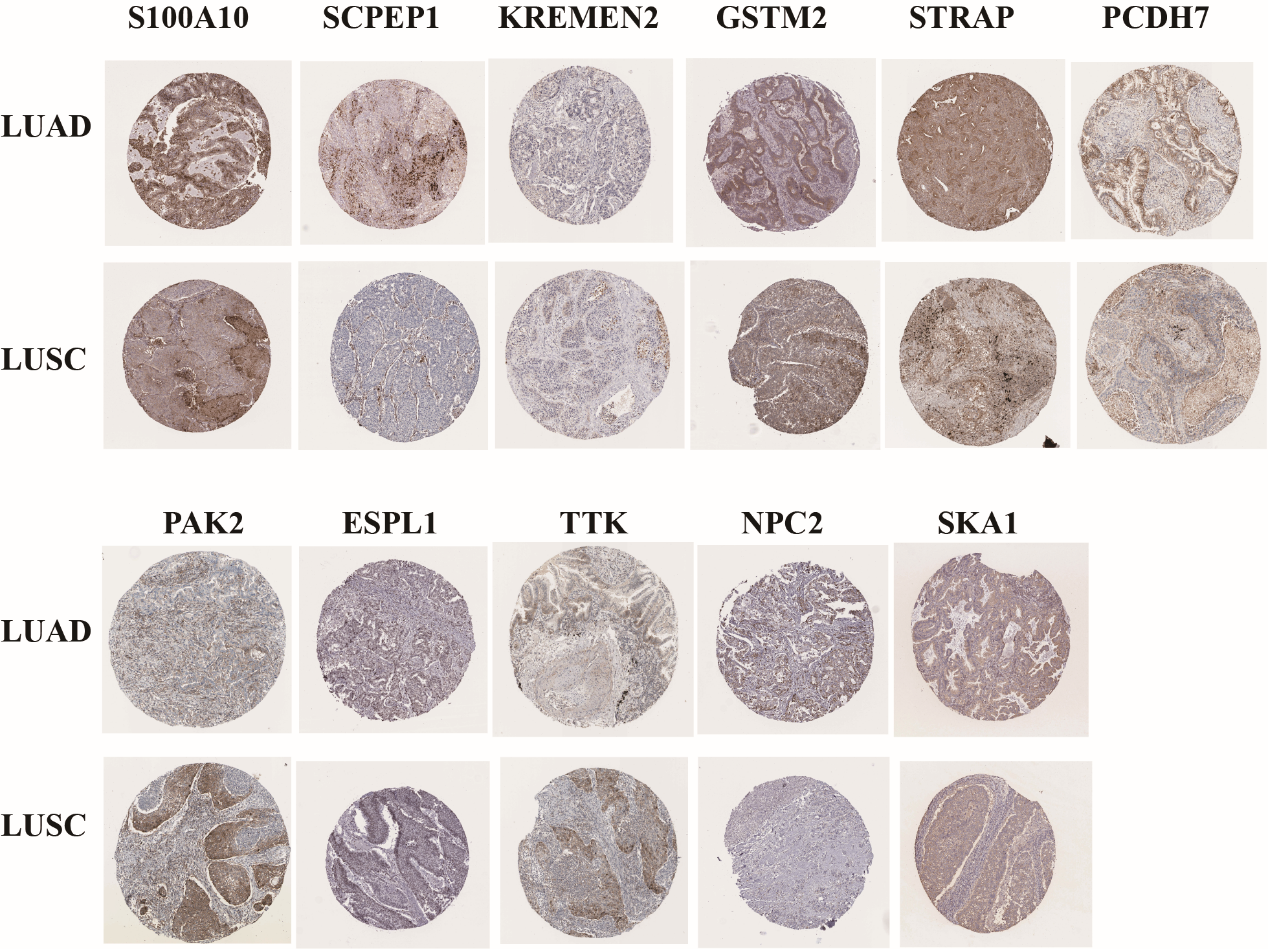

Supplement: S13 Fig — The protein expressions of S100A10, GSTM2, STRAP, PCDH7, PAK2, ESPL1, TTK, SKA1 were strongly positive in LUAD and LUSC tissues, The protein expression of NPC2 was strongly positive in LUAD tissues and negative in LUSC tissues. (TIF) [file pcbi.1014050.s013.tif]

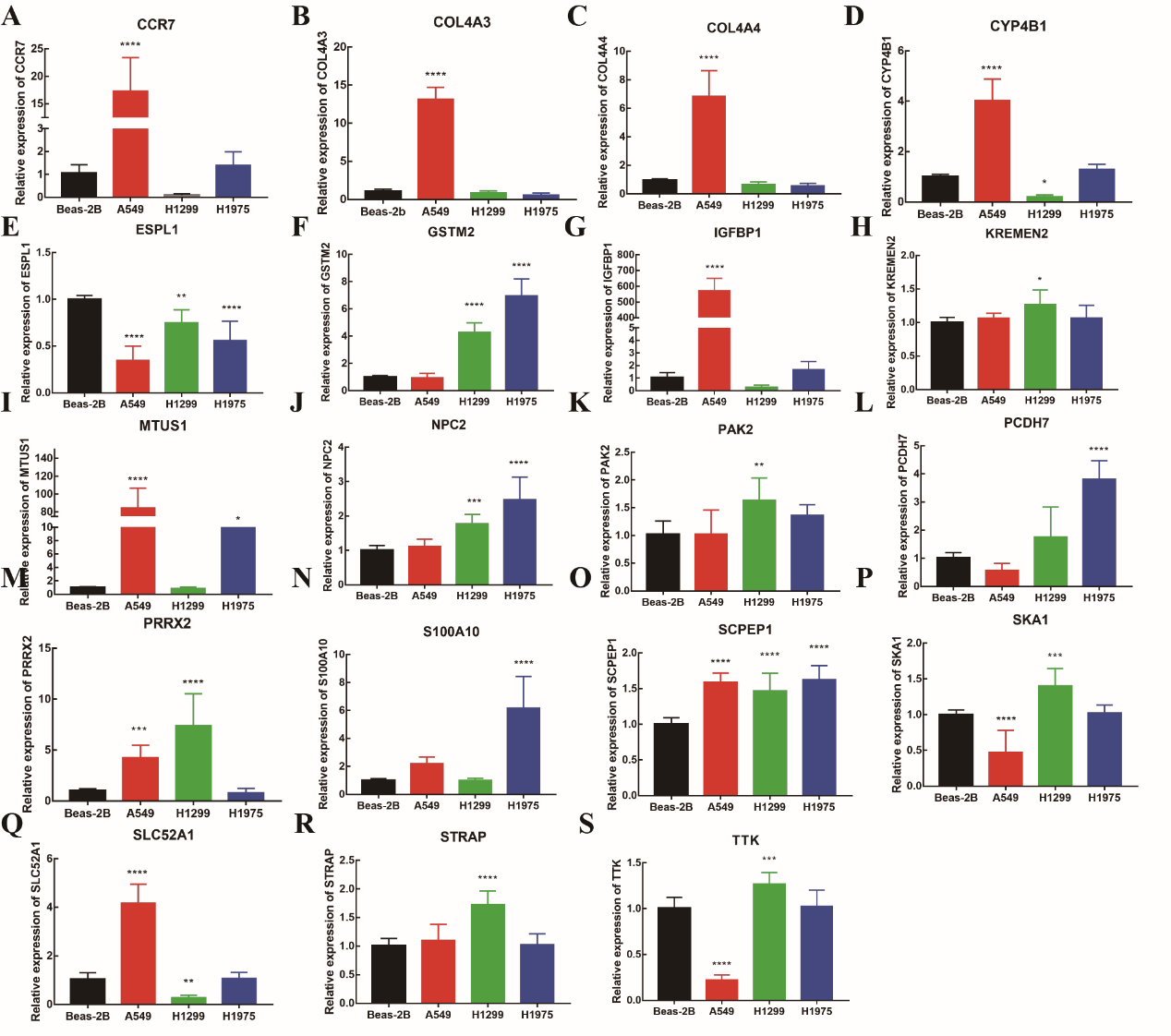

Supplement: S14 Fig — A-S The expressions of CCR7, COL4A3, COL4A4, CYP4B1, IGFBP1, MTUS1, SCPEP1, SLC52A1 were highly expressed in A549 cell lines. The expression of ESPL1, GSTM2, KREMEN2, NPC2, PAK2, PCDH7, PRRX2, SCPEP1, SKA1, STRAP, TTK were highly expressed in H1299 cell lines. The expressions of ESPL1, GSTM2, MTUS1, NPC2, PCDH7, S100A10, SCPEP1, SKA1 were highly expressed in H1975 cell lines. * Represents P < 0.05, ** represents P < 0.01, *** represents P < 0.001, **** represents P < 0.0001. (TIF) [file pcbi.1014050.s014.tif]

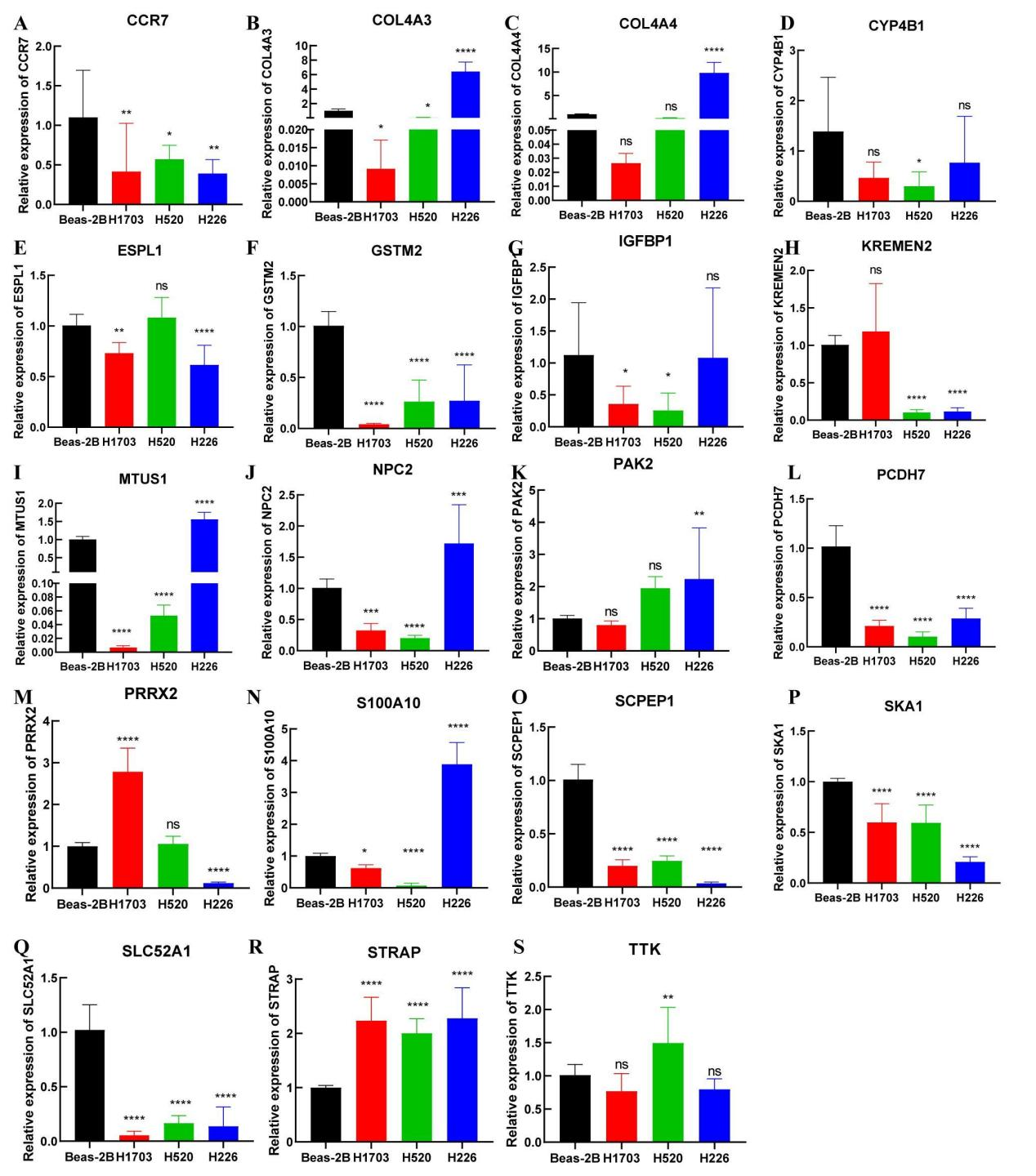

Supplement: S15 Fig — A-S The expressions of CCR7, COL4A3, ESPL1, GSTM2, IGFBP1, MTUS1, NPC2, PCDH7, S100A10, SCPEP1, SKA1, SLC52A1 were down-expressed in H1703 celll lines. The expressions of PRRX2, STRAP were highly expressed in H1703 cell lines. The expressions of CCR7, COL4A3, CYP4B1, GSTM2, IGFBP1, KREMEN2, MTUS1, NPC2, PCDH7, S100A10, SCPEP1, SKA1, SLC52A1 were down-expressed in H520 cell lines. The expressions of STRAP, TTK were highly expressed in H520 cell lines. The expressions of CCR7, ESPL1, GSTM2, KREMEN2, PCDH7, PRRX2, SCPEP1, SKA1, SCL52A1 were down-expressed in H226 cell lines. The expressions of COL4A3, COL4A4, MTUS1, NPC2, PAK2, S100A10, STRAP were highly expressed in H226 cell lines. * Represents P < 0.05, ** represents P < 0.01, *** represents P < 0.001, **** represents P < 0.0001. (TIF) [file pcbi.1014050.s015.tif]

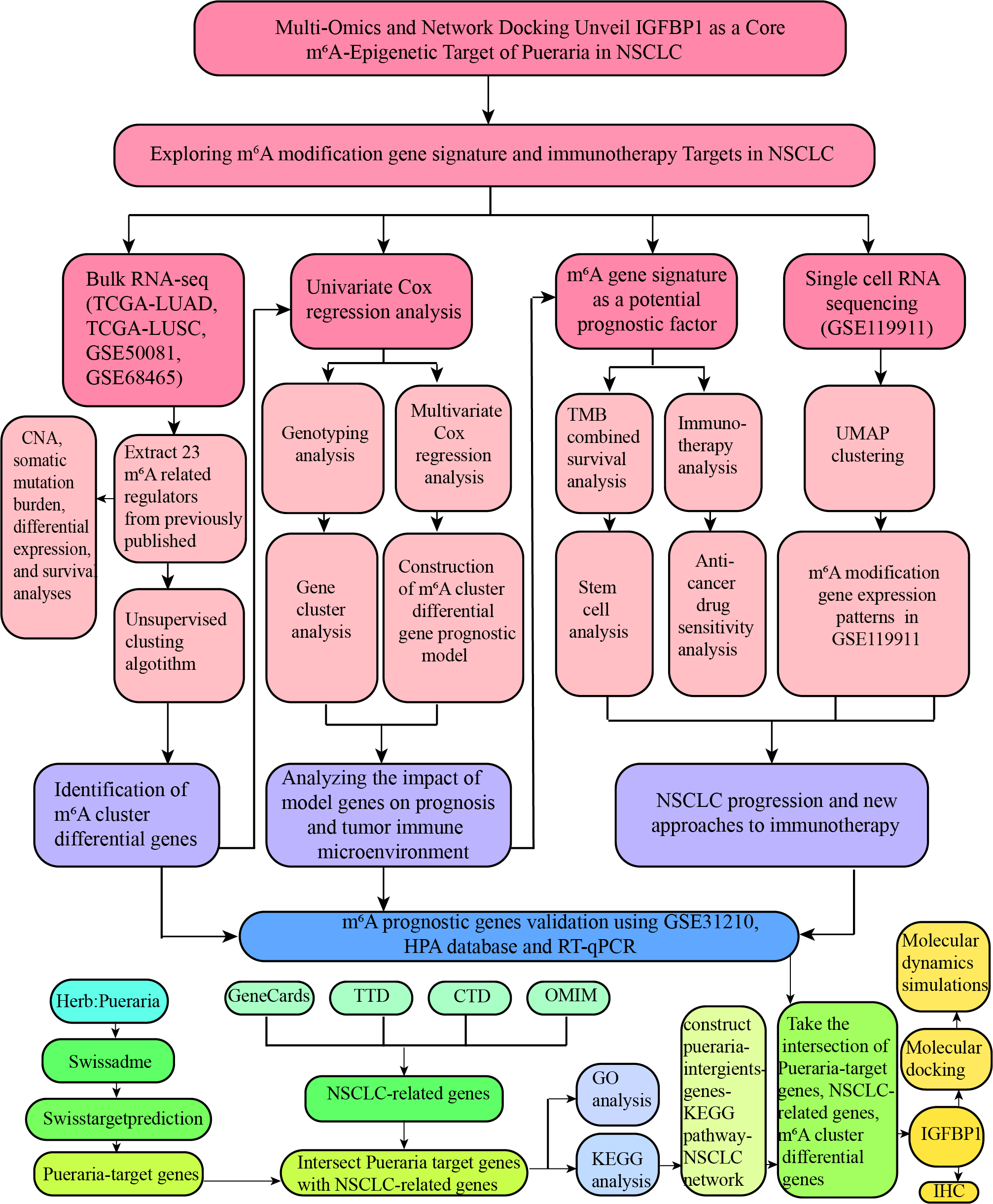

Supplement: S16 Fig — The technology diagram of the whole study. (TIF) [file pcbi.1014050.s016.tif]
