# Supplementary material for: Multi-omics and network pharmacology identify IGFBP1 as an m6A-Epigenetic target of pueraria in NSCLC therapy
Source: PLoS Comput Biol. 2026 Mar 12;22(3):e1014050. doi: 10.1371/journal.pcbi.1014050 (PMC12981440; doi:10.1371/journal.pcbi.1014050)
Supplement: Attachment — Submitted filename: Response to Reviewers.docx [file pcbi.1014050.s021.docx]

Additional Editor Comments:

Please find below the comments and suggestions provided by the invited reviewers to assist you in preparing the revised version of your manuscript. I believe that your study is both valuable and relevant for publication in PLOS Computational Biology.

In your revision, please ensure that the aims and hypotheses are clearly stated, the figure numbering and corresponding references in the main text are consistent, and all acronyms are properly defined. Additionally, I encourage you to discuss your main findings more thoroughly, improve the logical organization of the Results section, and expand the Methods section to include details, eg, in the molecular dynamics (MD) setup, equilibration procedures, production runs, and analysis protocols.

Responses: Thanks for your comments. We have clearly stated the aims and hypotheses of our study in the revised manuscript to ensure better clarity and focus. The figure numbering has been checked and corrected, and all corresponding references in the main text have been updated for consistency. All acronyms have been properly defined upon first use in the revised manuscript to enhance readability. We have expanded the discussion of our main findings to provide a more thorough interpretation and context. The results section has been reorganized for better logical flow, with clearer connections between experimental data and interpretations. The methods section has been expanded to include detailed descriptions of the molecular dynamics setup, equilibration procedures, production runs, and analysis protocols, ensuring reproducibility of our work.

Reviewer #1: Dear Authors

The research is informative and shows a strong computational and bioinformatics workflow (clustering, survival, mutation, CNV, immune infiltration, and pathway analysis). It reflects an advanced use of multi-omics data from TCGA and GEO.

However, the text has conceptual and methodological gaps, and the structure lacks clarity for publication in a high-impact journal.

Major Weaknesses

Lack of Clear Objective

The text jumps directly into results (m6A regulators, LUAD/LUSC analysis) without stating the central hypothesis or specific research question.

It’s unclear whether the study aims to identify prognostic markers, immune subtypes, or therapeutic targets.

Responses: Thanks for your comments. We appreciate your feedback regarding the clarity of our study's objective. In the revised manuscript, we have included a clear statement of our central hypothesis and specific research questions in the abstract and introduction section. We explicitly state that the aim of our study is to identify prognostic markers and therapeutic targets related to m^6^A regulators in NSCLC. This addition should provide a clearer context for the results presented in the subsequent sections. (Page 2, lines 26-28; Page 5, lines 104-107).

Missing Methodological Details

No information on dataset size, normalization methods, statistical thresholds (e.g., adjusted p-values, FDR), or the exact bioinformatics pipeline.

Responses: Thanks for your comments. We appreciate your feedback regarding the methodological details. In the revised manuscript, we have included comprehensive information about the dataset size, normalization methods, and statistical thresholds. Specifically, we have detailed the size of the dataset used in our analysis, the normalization techniques applied, and the adjusted p-values and false discovery rate (FDR) thresholds that were utilized throughout the study. Additionally, we have provided a clear description of the bioinformatics pipeline, outlining each step taken in our analysis (Pages 5-14).

The basis for clustering (consensus clustering parameters, number of clusters k, etc.) is not explained.

Responses: Thank you for pointing this out. We have now included a detailed explanation of the consensus clustering parameters used in our analysis, including the rationale behind the selection of the number of clusters (k). This information has been added to the methods section to ensure clarity and reproducibility of our clustering approach (Page 6, lines 125-132; Page 7, lines 133-140; Page 8, lines 169-175).

No Validation

There’s no mention of experimental validation (e.g., RT-qPCR, IHC, or external dataset verification).

Computational results alone may not confirm biological relevance.

Responses: Thanks for your comments. We acknowledge the importance of experimental validation. In the revised manuscript, We have validated the expression levels of the m^6^A prognostic genes in NSCLC cell lines using RT-qPCR in our manuscript (Supplementary Figures 14-15). Additionally, immunohistochemistry (IHC) was employed to confirm the expression levels of the key m^6^A gene IGFBP1 in NSCLC tissues from 10 cases of LUAD and 10 cases of LUSC that we collected (Figure 8). This expression aligns with the tissue-level expression found in LUAD and LUSC patients in the TCGA database. Furthermore, we have used external dataset GSE31210 that support our findings, highlighting the biological relevance of our computational results. We believe that these additions strengthen the manuscript and address your concerns regarding validation (Page 11, lines 221-242; Page 12, line 243; Page 18, lines 394-396; Page 19, lines 397-399; Page 24, lines 517-528; Page 25, line 529; Page 26, lines 567-572; Page 27, lines 573-579).

Overgeneralized Conclusions

Claims about immune microenvironment regulation and clinical relevance are strong but not supported by mechanistic evidence.

Responses: Thanks for your comments. We appreciate your feedback regarding the conclusions drawn in our manuscript. In response to your concern, we have revised the discussion section to clarify the limitations of our findings and to emphasize the need for further mechanistic studies. We have also included additional references to support our claims about immune microenvironment regulation, ensuring that our conclusions are more accurately framed in the context of existing literature. This should address the concern regarding the strength of our claims and provide a more balanced view of the clinical relevance of our findings (Page 30, lines 651-660; Page 31, lines 661-662).

No discussion of potential confounders or limitations.

Responses: Thanks for your comments. We appreciate your feedback regarding the discussion of potential confounders and limitations. In the revised manuscript, we have added a dedicated section that outlines the potential confounders that could impact our results, including demographic variables and external factors. Additionally, we have discussed the limitations of our study, such as sample size and generalizability, to provide a more comprehensive understanding of the findings. Thank you for bringing this important aspect to our attention (Page 31, lines 679-682; Page 32, lines 690-695 ).

Figure / Table References Missing

The text refers to results (e.g., subtypes, immune infiltration) but provides no figure or table references, which weakens reproducibility.

Responses: Thanks for your comments. We appreciate your feedback regarding the missing figure and table references. In the revised manuscript, we have added appropriate figure and table references throughout the text to enhance clarity and reproducibility. Each result discussed, including subtypes and immune infiltration, is now clearly linked to its corresponding figure or table, ensuring that readers can easily locate the relevant data.

Weak Link to Clinical Translation

Although clinical outcomes (survival) are mentioned, there is no clear suggestion on how identified m6A regulators could be applied in diagnosis or treatment.

Responses: Thanks for your comments. We appreciate your feedback regarding the clinical translation of our findings. In the revised manuscript, we have included a dedicated section discussing the potential applications of identified m^6^A regulators in both diagnosis and treatment. Specifically, we have outlined how these regulators can serve as biomarkers for patient stratification and how targeting them could enhance therapeutic strategies in relevant cancer types. This addition aims to strengthen the link between our research findings and their clinical implications (Page 29, lines 636-638; Page 30, lines 639-643).

Language & Structure

Long, complex sentences reduce clarity.

Responses: Thanks for your comments. We have revised the manuscript to simplify long and complex sentences, enhancing clarity throughout the text. Each section has been carefully edited to improve readability and ensure that the main ideas are conveyed more effectively.

Some transitions between LUAD and LUSC analyses are abrupt.

Responses: Thanks for your comments. We have improved the transitions between the LUAD and LUSC analyses by adding transitional phrases and contextual information. This adjustment provides a smoother flow and better connects the discussions of the two analyses, making it easier for readers to follow the narrative.

Recommendations

Add a clear research aim and hypothesis.

Responses: Thanks for your comments. We have added a clear research aim and hypothesis in the introduction section of the manuscript. The research aim is now stated as: “To identify m^6^A-based prognostic subtypes in NSCLC.”The hypothesis posits that specific m^6^A modifications can serve as reliable biomarkers for prognosis in NSCLC patients (Page 5, lines 104-107).

Define what biological question the study answers (e.g., “to identify m6A-based prognostic subtypes in NSCLC”).

Responses: Thanks for your comments. We have explicitly defined the biological question addressed by our study in the introduction. The study aims to identify prognostic markers and therapeutic targets related to m^6^A regulators in NSCLC. thereby providing insights into the molecular mechanisms underlying the disease and its progression.(Page 2, line 26-28; Page 5, line 104-107).

Expand the Methods section.

Describe all data sources, preprocessing steps, and statistical thresholds.

Include package names, software versions, and cut-off criteria for DEGs or clustering.

Include experimental or external validation.

Responses: Thanks for your comments. We appreciate your suggestions and have updated the methods section in the revised manuscript accordingly. (Pages 5-14 ).

Validate expression of key m6A genes via RT-qPCR or IHC.

Responses: Thanks for your comments. We appreciate your suggestion and have added the validation of IGFBP1 expression in NSCLC tissues using IHC in the revised manuscript according to your suggestions (Page 11, lines 233-242; Page 12, line 243; Page 26, lines 567-572; Page 27, lines 573-579; Figure 8).

Use an independent GEO dataset for model verification.

Responses: Thank you for your comments. According to your suggestion, we have added an independent GEO dataset (GSE31210) to validate the model in the revised manuscript. (Page 6, lines 119-123; Page 7, lines 148-154; Page 8, lines 155-161; Page 18, lines 394-396; Page 19, lines 397-399; Figure 1T).

Reorganize Results logically.

(a) Subtype identification →

(b) Mutation/CNV landscape →

(c) Immune infiltration →

(d) Prognostic analysis →

(e) Pathway enrichment.

Add visual evidence.

Figures showing Kaplan–Meier survival curves, heatmaps, immune scores, and m6A regulator expression patterns.

Responses: Thanks for your comments. We appreciate your suggestion and have revised the presentation of the results accordingly in the updated manuscript. (Pages 14-28).

Strengthen Discussion.

Compare findings with previous studies on m6A in NSCLC.

Address limitations (e.g., reliance on public data, lack of experimental confirmation).

Improve English clarity.

Shorten sentences, avoid redundancy, and use transition phrases (“In contrast…”, “Furthermore…”).

Responses: Thanks for your comments. We have revised the Discussion section to provide a more comprehensive analysis of our findings. We included additional context and implications of our results, emphasizing their significance in the field of m^6^A research in NSCLC. We have incorporated a comparison of our findings with previous studies on m^6^A in NSCLC ( Page 31, lines 676-679). This includes a detailed analysis of how our results align or differ from existing literature, highlighting the contributions of our study to the current understanding of m^6^A modifications in NSCLC. We have explicitly addressed the limitations of our study in the revised manuscript (Page 32, lines 690-695). We have thoroughly revised the manuscript to enhance the clarity of the English language. This includes correcting grammatical errors and improving overall readability. We have shortened sentences and eliminated redundancy throughout the manuscript. Additionally, we have incorporated transition phrases such as “In contrast…” and “Furthermore…” to improve the flow of the text and enhance the coherence of our arguments.

Provide a concise conclusion.

Emphasize the novelty and practical implications (e.g., “m6A-related genes may guide immunotherapy response in NSCLC”).

Responses: Thanks for your comments. We appreciate your suggestion and have updated the conclusion in the manuscript accordingly (Page 32, lines 696-702).

Reviewer #2: This is an interesting research manuscript by Rui Li and co-workers on a topic of growing importance in the current oncotherapy landscape titled Multi-Omics and Network Pharmacology Identify IGFBP1 as an m6A-Epigenetic Target of Pueraria in NSCLC Therapy.

Overall, the strategic approach was novel in the manuscript. I am supporting the publication of this article in PLOS Computational Biology. However, before this is possible, this article will need some serious revisions.

The authors reported the Identification of targets for cancer. The dysregulation of N6-methyladenosine (m6A) modification drives progression in non-therapeutics

Few of the cases author needs to modify the author grammatical errors.

Responses: Thanks for your comments. We have thoroughly reviewed the manuscript and corrected all identified grammatical errors to enhance the clarity and readability of the text. We have made revisions to clarify our methodology and the significance of our findings, ensuring that the strategic approach is well-articulated throughout the manuscript.

Based on the sequence analysis, MD Simulation, Docking reviewd suggest that some merit in the paper.

Responses: Thanks for your comments. We have expanded the discussion around our sequence analysis, MD simulation, and docking studies to highlight their significance and the insights they provide into the interactions and mechanisms involved in NSCLC therapy (Page 13, lines 281-286; Page 14, lines 287-292).

This is an interesting research manuscript by Rui Li and co-workers on a topic of growing importance in the current oncotherapy landscape titled Multi-Omics and Network Pharmacology Identify IGFBP1 as an m6A-Epigenetic Target of Pueraria in NSCLC Therapy.

Originality: Excellent

Technical Quality: Good

Clarity of Presentation: Fair

Responses: Thanks for your comments. We have revised the manuscript to improve the clarity of presentation by restructuring sentences, enhancing the flow of information, and ensuring that complex concepts are explained more clearly.

Rate the overall importance of this paper to the field of Medicinal Chemistry: Top 10%

Recommended after minor revision
